# CD160 Plays a Protective Role During Chronic Infection by Enhancing Both Functionalities and Proliferative Capacity of CD8+ T Cells

**DOI:** 10.3389/fimmu.2020.02188

**Published:** 2020-09-11

**Authors:** Linxia Zhang, Anli Zhang, Jun Xu, Chao Qiu, Lingyan Zhu, Chenli Qiu, Weihui Fu, Ying Wang, Lilin Ye, Yang-xin Fu, Chen Zhao, Xiaoyan Zhang, Jianqing Xu

**Affiliations:** ^1^Shanghai Public Health Clinical Center and Institutes of Biomedical Sciences, Shanghai Medical College, Fudan University, Shanghai, China; ^2^Department of Pathology, University of Texas Southwestern Medical Center, Dallas, TX, United States; ^3^Department of AIDS/STD, Shanghai Municipal Center for Disease Control and Prevention, Shanghai, China; ^4^Institute of Immunology, Army Medical University, Chongqing, China

**Keywords:** HIV-1, chronic infection, protective immunity, T cell, CD160

## Abstract

The understanding of protective immunity during HIV infection remains elusive. Here we showed that CD160 defines a polyfunctional and proliferative CD8+ T cell subset with a protective role during chronic HIV-1 infection. CD160+ CD8+ T cells derived from HIV+ patients correlated with slow progressions both in a cross-sectional study and in a 60-month longitudinal cohort, displaying enhanced cytotoxicity and proliferative capacity in response to HIV Gag stimulation; triggering CD160 promoted their functionalities through MEK–ERK and PI3K–AKT pathways. These observations were corroborated by studying chronic lymphocytic choriomeningitis virus (LCMV) infection in mice. The genetic ablation of CD160 severely impaired LCMV-specific CD8+ T cell functionalities and thereby resulted in loss of virus control. Interestingly, transcriptional profiling showed multiple costimulatory and survival pathways likely to be involved in CD160+ T cell development. Our data demonstrated that CD160 acts as a costimulatory molecule positively regulating CD8+ T cells during chronic viral infections, thus representing a potential target for immune intervention.

## Introduction

It is known that CD8+ cytotoxic T lymphocytes play a critical role in the containment of virus replication during HIV-1 and SIV infections, despite being incapable of preventing the progression to AIDS in most cases (1). During acute HIV-1 infection, proliferative and polyfunctional CD8+ T cells are elicited to mount an effective attack on infected cells (2). However, in the case of persistent HIV-1 infection, CD8+ T cells lose their functions in a hierarchical manner, successively impaired in producing IL-2, proliferating, delivering cytotoxic molecules like perforin and granzymes, producing TNF, IFN-γ, and MIP-1β, and finally attaining a so-called exhausted state (3). Although the exact mechanisms involved have not been clarified in detail, known co-inhibitory receptors such as PD-1, CTLA-4, Tim-3, and LAG-3 have been proposed to contribute to the exhaustion of T cells, as they were specifically accumulated in HIV-1-specific T cells (3, 4). Moreover, long-term non-progressors express lower levels of PD-1 than typical progressors (TP) (5, 6). Consequently, single/combined blockade of co-inhibitory receptor has been attempted to restore T cell function in both SIV- and HIV-1-infected models and has shown promising results (6–12).

CD160, initially identified as a glycosylphosphatidylinositol-anchored cell surface protein with an IgV-like domain in the extracellular region, is expressed on multiple immune cells, including intestinal intraepithelial T lymphocytes, CD56^*dim*^CD16+ NK lymphocytes, and a minor subset of CD4+ T cells and CD8+ T cells (13). CD160 has two known interaction partners including MHC class I (MHC-I) (14) and herpes virus entry mediator (HVEM) (15), with potential to transduce signaling both as a ligand and a receptor. It has been speculated that MHC-I, when triggered by CD160 ligation, transduces a costimulatory signal to compensate the loss of CD28 in certain CD28−/− lymphocyte types, such as intestinal intraepithelial T lymphocytes (16, 17). CD160 binds to HVEM with a significantly higher affinity than MHC-I (18). The CD160–HVEM network was found to deliver a co-activating signal to NK cells (19) while playing an inhibitory role in regulating CD4+ T cells (15) and NKT cells (20).

CD160 is generally perceived as a co-inhibitory receptor contributing to the erosion of CD8+ T cell responses in chronic viral infection. However, how CD160 tunes the CD8+ T cell functions during chronic antigen stimulation remains ambiguous. In this study, we determined the roles of CD160 in CD8+ T cell response in two chronic infections, HIV in human and LCMV in mice. Our results established CD160 as a costimulatory molecule on CD8+ T cells and with a protective role during chronic infection.

## Materials and Methods

### Human Subjects

Blood samples were collected from cohorts of HIV-1-clade-B-infected slow progressors (SP, defined as subjects with peripheral absolute CD4 count >400 cells/μl and plasma viral load <5,000 copies/ml for at least 8 years) and typical progressors (TP, defined as subjects with viral load >5,000 copies/ml and peripheral absolute CD4 count <350 cells/μl) from Shan-Xi Province, PR China (Supplementary Table S1). All the patients were treatment naïve. Eleven of them initiated antiretroviral therapy (ART) during our 3-year-follow up. Age- and gender-matched HIV-seronegative blood donors were recruited from Fudan University, Shanghai, China.

### Mice, Virus, and Infections

The C57BL/6J mice (H-2D^b^) were from Jackson Laboratories. The CD160KO mice were provided by Y.X. Fu (19) (University of Texas Southwestern Medical Center). The P14 (CD90.1+ CD90.2+) TCR transgenic mice were provided by Dr. R. Ahmed (Emory University). The mice used in the experiments were 6 to 12 weeks old and gender-matched and maintained under specific pathogen-free conditions. Lymphocytic choriomeningitis virus (LCMV) Clone 13 strain, provided by Dr. R. Ahmed (Emory University), was propagated in BHK21 cells and titrated in VERO cells using immune focus assay with anti-LCMV antibody (Clone: M104, Abcam).

### Flow Cytometry and Antibodies

The antibodies and other reagents are listed in Supplementary Information. Surface staining was performed as described previously (12). For intracellular cytokine production analyses, human peripheral blood mononuclear cells (PBMCs) or mouse splenocytes were stimulated with the indicated peptide (0.5 μg/ml) in the presence or the absence of 10 μg/ml of the indicated antibody, including CL1R2 antibody (anti-CD160, clone: CL1-R2, MBL) and mIgG (BioLegend, clone: MOPC-173). At 1 h later, Brefeldin A (Sigma, 2 μg/ml) and Monensin (Sigma, 2 μM) were added, followed by incubation for 5 h at 37°C. After surface staining and near-IR staining for viability, the cells were permeabilized with Cytofix/Cytoperm Fixation/Permeabilization kit (BD Biosciences) according to the manufacturer’s instructions and stained with fluorescently labeled antibodies against granzyme B, CD107a, IFN-γ, and TNF-α. Flow cytometry and cell sorting were respectively performed on Fortessa and FACSAria, and the data were analyzed using FlowJo software (Tree Star).

### ELISPOT and Proliferation Assays

For the enzyme-linked immune absorbent spot (ELISPOT) assay, 2 × 10^5^ purified CD8+ T cells were seeded in Granzyme B ELISPOT plates (R&D) and stimulated with HIV-1 clade B Gag pooled peptides (B Gag) (2.5 mg/ml, National Institutes of Health AIDS Research and Reference Reagent), with the presence of 10 μg/ml of mIgG control or CL1R2 antibody, for 24 h. The plates were then washed and processed according to the manufacturer’s protocol. ChampSpot IV Bioreader (Beijing SAGE Creation Science, Beijing, China) ELISPOT system was used for data analysis. The proliferation of sorted CD160± CD8+ T cells was assessed by Alexa670 dye (Invitrogen) dilution. Briefly, phosphate-buffered saline (PBS)-resuspended cells (1 × 10^7^/ml) were incubated with 5 uM of Alexa670 dye at 37°C for 8 min before the reaction was stopped by adding 1/5 volume of FBS prior to incubation on ice for 5 min. After washing with PBS, the labeled cells were seeded at 2 × 10^5^ per 96-well plate, stimulated with Gag peptides for the indicated days, and then analyzed by flow cytometry.

### Adoptive Transfer

CD8+ T cells were isolated from spleens and lymphoid node of naive wild typeWT P14 (CD90.1+ CD90.2+) or CD160KO P14 (CD90.1+ CD90.2−) mice by CD8+ T cell negative selection (STEMCELL Technologies). The indicated numbers of WT or CD160KO P14 CD8+ T cells were mixed at a 1:1 ratio and transferred into naive recipient (CD90.1−CD90.2+) mice. The mice were infected with LCMV Clone 13 on the next day.

### Virus Titration and Quantification

For LCMV Clone 13 infection, the mice were intravenously infected (2 × 10^6^ pfu/mice) and then were housed in a biosafety-level-2 facility. The LCMV viral loads in serum and organs were quantified by real-time PCR assay. The primers used were LCMV GP forward primer 5′-CATTCACCTGGACTTTGTCAGACTC-3′ and LCMV GP reverse primer 5′-GCAACTGCTGTGTTCCCGAAAC-3′ (21).

### Transcriptome Profiling and Analysis

RNA was isolated from freshly isolated tetramer+CD160+ CD8+ T cells and tetramer+CD160-CD8+ T cells using TRIzol coupled with ZYMO RNA extraction kit. Microarray analyses were performed with Agilent Mouse Gene Expression Chip (8^∗^60K, design ID: 028005). The log2-transformed data were normalized by median normalization. Genes that differentially expressed over 1.5-fold change, with threshold *p* value < 0.05, were analyzed for functional enrichment using DAVID Bioinformatics^1^ and hierarchical clustering analysis carried out with Biometric Research Branch-ArrayTools version 4.1.0 beta 2 (22, 23).

### Immunoblotting and Real-Time PCR

For lysate preparation, 1–1.5 × 10^6^ primary CD8+ T cells were resuspended in 100 μl of PBS, followed by incubation with 10 μg/ml mIgG or CL1R2 antibody for 30 min at 37°C. The cells were then treated with magnetic beads conjugated with anti-CD3 antibodies. Samples were collected at 0.5, 2, 10, 30, and 60 min after for immunoblotting analysis with the indicated antibody, and the protein bands were visualized using Odyssey^®^ Fc Imaging System (LI-COR Biotechnology). The antibodies specific for pAKT(Ser473) (D9E), pERK(Thr202/Tyr204) (D13.14.4E), and ERK1/2(137F5) were from Cell Signaling Technology, and anti-β-actin antibody (AC-15) was from Santa Cruz. For real-time PCR analysis, RNA samples were prepared by lysing cells with RNAzol reagent (MRC) followed by purification with an RNA extraction kit (ZYMO). RNA was reverse-transcribed using Reverse Transcription System (Promega), and cDNA quantification was then performed using SYBR Green RT-PCR kit (Promega) on a Real-Time PCR System (Eppendorf 7500).

### Statistical Analyses

Analyses were conducted using Grand Prism 7 software (San Diego, CA, United States). Mann–Whitney *U* test was utilized to determine the significant differences between subject groups. Paired *t* test was employed for comparisons between matched groups. Data were expressed as means ± SEM. Spearman correlation test was performed to analyze the associations between CD160/PD1 expression and CD4 counts or viral loads. Log-rank test was used for survival prediction analysis. Values of *p* < 0.05 were considered significant.

### Study Approval

Animal experiments were conducted with the approval of the Institutional Animal Care and Use Committee of Shanghai Public Health Clinical Center (SPHCC). Written informed consents were provided by all the participants. The overall study was reviewed and approved by the Ethics Committee of SPHCC.

## Results

### CD160 Correlates With Slow Progression During Chronic HIV-1 Infection

**FIGURE 1:**
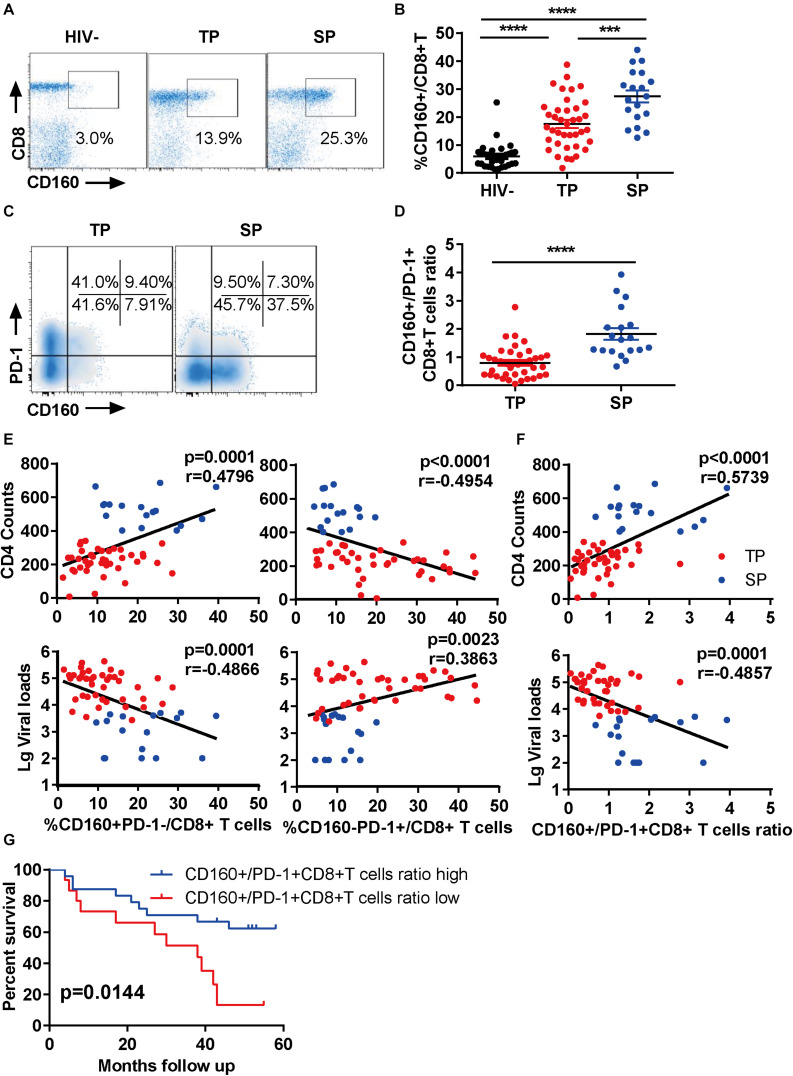
CD160 correlates with slow progression during chronic HIV-1 infection. **(A,B)** Fresh human peripheral blood mononuclear cells were stained for CD160 expression on CD8+ T cells from three groups: slow progressors (SP), typical progressors (TP), and HIV-1 seronegative controls (HIV-). Representative flow cytometric plots are shown in **(A)** and summarized data in **(B)**. SP, *n* = 19; TP, *n* = 39; HIV-, *n* = 33. **(C,D)** Relative expression of CD160 and PD-1 on CD8+ T cells in SP *versus* TP. Representative flow cytometric plots are shown in **(C)** and summarized data in **(D)**. **(E)** Association between CD160+ PD-1- *versus* CD160-PD-1+ CD8+ T cell subpopulation and CD4 counts (upper panel) or plasma viral loads (lower panel) in HIV-1-infected subjects. **(F)** Association between relative CD160+/PD-1+ frequency and CD4 counts (upper panel) or plasma viral loads (lower panel) in HIV-1-infected subjects. SP, *n* = 17; TP, *n* = 41. **(G)** Prediction of disease progression using the ratio of CD160+ subpopulation to PD-1+ subpopulation with 5 years of follow-up. *n* = 56. The data in **(B,D–G)** are analyzed by Mann–Whitney *U* test, Spearman correlation, and log-rank test, respectively. The calculated *p* and *r* values are presented in the corresponding figures. The error bars in **(B,D)** represent SEM. ***P < 0.001; ****P < 0.0001.

To explore the function of CD160 in CD8+ T cells during chronic HIV-1 infection, we first examined the enrichment of CD160-positive CD8+ T cells from three groups (Supplementary Table S1): HIV-1-infected slow progressors (SP), HIV-1-infected typical progressors (TP), and HIV-seronegative individuals. As expected, we observed an elevation of CD160+ CD8+ T cell frequencies in HIV-1-infected individuals compared with HIV-seronegative controls (Figures 1A,B). Intriguingly, the frequency of CD160+ CD8+ T cells was further elevated in SP than in TP (Figure 1B), which was consistent with two previous reports (24, 25).

PD-1 has been shown to be up-regulated on CD8+ T cells during HIV-1 infection, and its high expression is a hallmark of exhausted T cells (26). Thus, we determined the co-expression of CD160 and PD-1 in our samples. Unlike previous reports (24), we found that CD160+PD-1+CD8+ T cells represented only a small fraction of total CD8+ T cells in both SP and TP groups (Supplementary Figure S1A), with the clear presence of CD160+ PD-1- and CD160-PD-1+ subsets. Notably, the SP group showed a significant expansion of CD160+ subset at the expense of PD-1+ subset when compared to the TP group (Figures 1C,D). Therefore, CD160 and PD-1 expression in CD8+ T cells were largely uncoupled in chronic HIV infections.

Next, we analyzed the association between CD160-expressing CD8+ T cells and HIV-1 disease progression in our cross-sectional study. Unexpectedly, the frequencies of CD160+ PD-1-CD8+ T cells significantly correlated with CD4 counts and reversely with HIV-1 plasma viral loads (Figure 1E, left), whereas CD160-PD-1+ CD8+ T cells displayed an opposite association (Figure 1E, right). Contrastively, no correlation was observed between the other two T cell subsets, CD160-PD1-CD8+ and CD160+ PD1+ CD8+ T cells, with CD4 and viral measures (Supplementary Figure S1B). Furthermore, we found that the ratio of CD160+ CD8+ T cells to PD-1+ CD8+ T cells was a better correlate of CD4 counts and HIV-1 plasma viral loads than CD160 and PD-1 frequencies alone (Figure 1F). We also analyze the correlation of CD160 and PD-1 expression with CD4 counts and viral loads within TP group alone. We observed a correlation pattern consistent with the overall analysis described above, although there was no statistical significance, which is possibly attributed to the small cohort size (Supplementary Figure S1C). We investigated next the correlation between CD160 and PD-1 expression and clinical outcomes. The endpoints in our study were defined by one of the three criteria, including an absolute CD4+ T cell count of <350 cells/μl, initiation of long-term ART, and progression to AIDS and death. During the 5-year follow-up period, the participants in the study showed divergent clinical outcomes, with 19 of 56 (33.9%) meeting the criteria to reach the endpoint (Supplementary Table S1). When splitting our cohort into two halves according to the ratio of the percentage of CD160+ CD8+ T cells to that of PD-1+ CD8+ T cells, the group with high CD160+/PD-1+ CD8+ T cells ratio showed a better survival potential than the counterpart with low CD160+/PD-1+ CD8+ T cells ratio (Figure 1G). These data implicated that CD160, as opposed to PD-1, may positively, rather than negatively, regulate CD8+ T cell response during HIV infection.

### CD160 Defines a Functional and Proliferative CD8+ T Cell Subset in Response to HIV Gag Stimulation

**FIGURE 2:**
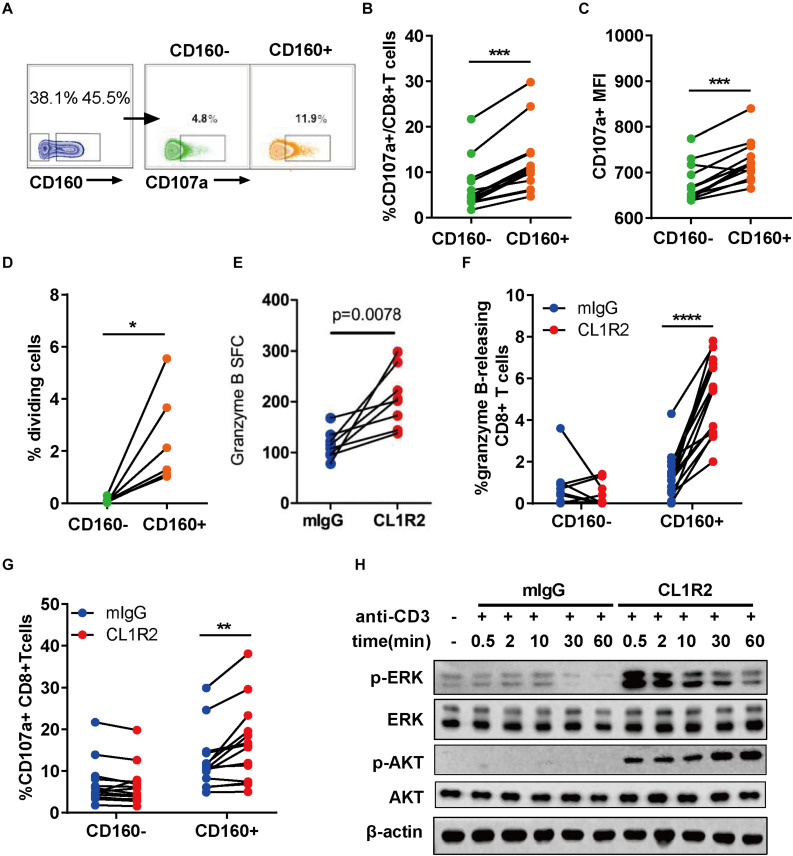
CD160 defines a functional and proliferative CD8+ T cell subset in response to HIV Gag stimulation. **(A)** Representative flow cytometric plots of gating strategy for the functional analyses of CD160– and CD160+ CD8+ T cells derived from patients with chronic HIV infection after HIV-1 Gag peptide stimulation. **(B,C)** Comparison of CD107a-expressing cell percentage **(B)** and mean fluorescence intensity of CD107a **(C)** between paired CD160– and CD160+ CD8+ T cells after HIV-1 Gag peptide stimulation, *n* = 14. **(D)** Assessment of HIV-1 Gag peptide-stimulated cell proliferation CD8+ T cells between CD160+ and CD160– T cells. The purified CD8+ T cells were labeled with efluor670 dye (5 uM) and then stimulated with HIV-1 Gag peptides for 7 days. Changes in the percentage of proliferating cells between paired CD160– and CD160+ CD8+ T are shown in **(D)** (*n* = 6). **(E)** Compiled data of ELISPOT assay determining the frequency of granzyme B-releasing cells within the total CD8+ T cells induced by HIV-1 Gag peptide stimulation in the presence of mIgG or CL1R2 antibody. Frequency is expressed as the number of cells per 10^6^ of total CD8+ T cells. *n* = 15. **(F,G)** Compiled data of flow cytometric analysis of the percentage change in granzyme B-releasing cells **(F)** and CD107a-positive cells **(G)** within the total CD8+ T cells induced by CL1R2 antibody relative to mIgG treatment, when both coupled with HIV Gag peptide stimulation. *n* = 15. **(H)** Immunoblotting analyses of time course of activation of AKT and ERK in human peripheral blood mononuclear cell-derived CD8+ T cells responding to the co-treatment of anti-CD3 antibody and CL1R2 antibody or mIgG. The abundance of phosphorylated AKT and ERK is normalized to that of β-actin and expressed as fold change relative to the values obtained with the samples exposed to anti-CD3 plus mIgG for 0.5 min. The data in **(B–G)** are analyzed by paired *t* test, with the *p* values indicated in the figure. *P < 0.05; **P < 0.01; ***P < 0.001; ****P < 0.0001.

To assess the functionality of CD160+ CD8+ T cells, we stimulated the PBMCs from ART-naïve HIV-1+ patients with HIV Gag peptides overnight. The expression of CD107a was then measured by flow cytometry using intracellular staining (27). The degranulation of CD160+ CD8+ T cells, as measured by both the frequencies of CD107a-positive cells and mean fluorescence intensity, was significantly greater than their CD160− counterparts (Figures 2A–C). The isotype control revealed no background staining (Supplementary Figure S2). Consistently, in both SP and TP groups, CD160 was enriched in TEM (CCR7-CD45RA-) and TEMRA (CCR7-CD45RA+) (28) when compared to naïve T cells (CCR7 + CD45RA+) or TCM (CCR7 + CD45RA-) (Supplementary Figures S3A,B). We further examined the co-distribution of CD160 and a series of well-defined T cell surface markers and found that CD160 was predominantly accompanied by HLA-DR and CD95 but only partially overlapped with CD127 and CD38 (Supplementary Figures S3C,D). Thus, CD160+ CD8+ T cells appear to constitute an activated cytotoxic CD8+ T cell subset.

Next, we examined the proliferative capacity of CD160+ and CD160− CD8+ T cells from HIV-1+ patients. After sorting, the two cell subpopulations were labeled with efluor670, followed by exposure to HIV Gag peptides. As shown in Figure 2D, CD160+ CD8+ T cells displayed a significantly higher proliferation than their CD160− counterpart that only showed a marginal one or even failure to do so. Of note is that the difference was significantly larger than that shown in the degranulation assay, firmly establishing HIV-specific CD160+ CD8+ T cells as a highly proliferative CD8+ T cell subset.

To determine whether CD160 is directly involved in regulating the functions of CD8+ T cells, we took advantage of CL1R2, an anti-CD160 antibody, to trigger CD160 signaling in couple with Gag-peptide stimulation (29). The levels of released granzyme B, as determined by ELISPOT assay, were significantly higher upon CL1R2 treatment than control mIgG treatment (Figure 2E). This was corroborated with the intracellular staining assay, which showed that CL1R2 significantly enhanced the responses of Granzyme B and CD107a-positive cells in CD160+ cells but failed to do so in CD160-CD8+ T cells (Figures 2F,G). Thus, for CD8+ T cells in chronic HIV infection, acquiring an optimal cytotoxicity appears to need CD160 signaling in addition to TCR engagement.

We subsequently sought to define the molecular nature of CD160 signaling pathway in CD8+ T cells. To this end, we stimulated CD8+ T cells with magnetic beads conjugated with monoclonal antibodies (mAbs) against CD3 that can directly activate TCR pathway, coupled with treatment of mIgG or CL1R2 antibody (Figure 2H). Under these conditions, we found that the MEK–ERK pathway and the PI3K–AKT pathway, two pathways with important roles in the proliferation and the activation of CD8+ T cells, were highly dependent on the engagement of CD160. As shown in Figure 2H, robust induction of phosphorylated ERK and AKT only occurred when the CL1R2 antibody co-administered with the anti-CD3 antibody. These results indicate a dual-activation effect of CD160 on the PI3K–AKT and MEK–ERK signaling pathways, in accordance with an augmented CD8+ T cell response.

### CD160 Marked a Highly Poly-Functional CD8+ T Cell Subset With Proliferative Potential During Chronic LCMV Infection in Mice

**FIGURE 3:**
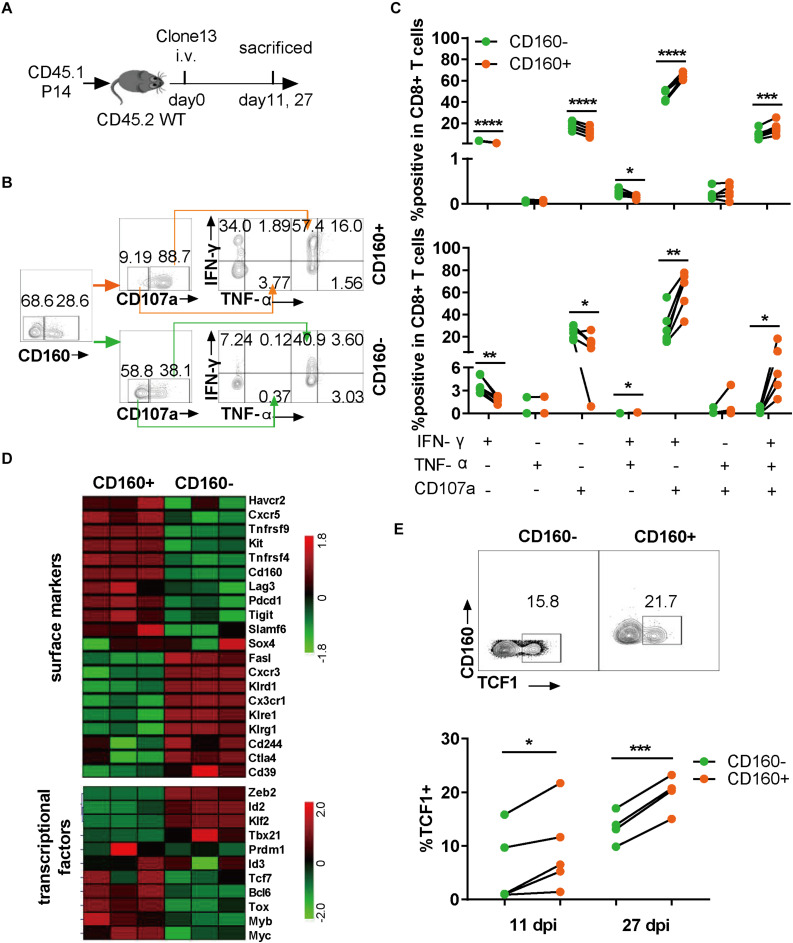
CD160 marked a highly poly-functional CD8+ T cell subset with proliferative potential during chronic lymphocytic choriomeningitis virus (LCMV) infection in mice. **(A)** Schematic diagram of using adoptive P14 CD8+ T cell transfer to study the CD160 effect in chronic LCMV infection of mouse. A total of 10^4^ of WT (CD45.1+) P14 CD8+ T cells were adoptively transferred into congenic (CD45.1–CD45.2+) naive recipient mice, which were subsequently infected with LCMV Clone 13. **(B)** Representative flow cytometric plots of gating strategy for analyzing the response of P14 cells isolated on 27 dpi to stimulation with GP33 peptide. **(C)** Comparison of magnitude and quality of responses of paired CD160– and CD160+ P14 T cells to GP33 peptide stimulation. The cells were analyzed on 11 dpi **(C)**, upper panel) or on 27 dpi **(C)**, lower panel). **(D)** Cell surface markers and transcriptional factors differentially expressed in LCMV-specific CD160– and CD160+ wild-type T cells as revealed by transcriptome profiling. CD8+ T cells from wild-type mice infected with LCMV Clone 13 for 7 days were stained with a mix of H-2D^b^ tetramer presenting LCMV-specific peptide (GP_33__–__41_, GP_276__–__286_, and NP_396__–__404_) and tetramer+CD160+ and CD160– T cells were sorted by flow cytometry for extracting RNA to perform microarray-based transcriptome profiling. Shown is the heatmap of detected differences in transcript levels of the indicated genes between CD160+ and CD160-CD8+ T cells. **(E)** Detection of TCF1 expression by flow cytometry on adoptively transferred P14 cells. Shown in the upper and the lower panels are representative flow cytometric plots (11 dpi) and pooled data of frequencies of TCF1-expressing cells, respectively. The experiments were independently repeated three times with *n* = 4–6 mice per experimental group. The data in **(C,E)** are analyzed by paired *t* test. *P < 0.05; **P < 0.01; ***P < 0.001; ****P < 0.0001.

As CD160 is conserved between human and mouse (13), we extended our research to chronic LCMV infection in mice, which allows a more precise dissection of CD160 function during the development of CD8+ T cell exhaustion. To exclude the effect of TCR specificity, we adoptively transferred CD8+ T cells from P14 mice, which carried an engineered TCR specifically recognizing the H-2D^b^-restricted GP33 epitope derived from LCMV glycoprotein (amino acids 33–41; KAVYNFATC), into congenic naive mice, followed by infection with LCMV Clone 13 (Figure 3A). The splenocytes were analyzed for their responses to GP33 peptide stimulation (Figure 3B) at either 11 or 27 days post-infection (dpi). We assessed the capability of CD8+ T cells to produce IFN-γ, TNF-α, and CD107a. As shown in Figure 3C, after GP33 peptide stimulation, both IFN-γ/CD107a dual- and IFN-γ/TNF-α/CD107 triple-functional responses displayed in CD160+ P14 T cells were significantly higher than that in their CD160− counterparts, whereas the difference in mono-functional response was largely negligible (Figure 3C). The superiority of CD160+ subset over CD160− subset in mounting poly-functional responses was further highlighted at 27 dpi (Figure 3C, lower panel). Thus, like in human, CD160 defines a potent poly-functional CD8+ T cell during chronic infection in mice.

To explore the mechanism by which CD160 regulated CD8+ T cell responses in chronic LCMV infection, we sorted virus-specific CD160+ tetramer+CD8+ T cells (CD160+) and CD160-tetramer+CD8+ T cells (CD160−) from splenocytes at 7 dpi and examined the gene expression difference between the two cell subpopulations by microarray profiling. A total of 2,593 genes were identified to be differentially expressed between CD160+ and CD160− cells, suggesting that they represent distinct CD8+ T cell subsets. Hierarchical clustering analysis subsequently revealed substantial differences in the expression of surface receptors and transcription factors between CD160+ and CD160− cells (Figure 3D). CD160+ cells showed selective expression of a number of co-stimulatory molecules such as *Tnfrsf4* (encoding OX-40) and *Tnfrsf9* (encoding 4-1BB), whereas CD160− cells preferentially expressed *Klrg1*, a hallmark of terminally differentiated CD8+ T cells. Within the transcription factor category, CD160+ CD8+ T cells had higher levels of *Bcl6*, *Tcf7* (encoding TCF1), *Bcl6*, *Myb*, and *Tox*, members of a transcriptional program promoting T cell proliferation and/or survival, but displayed lower abundance of *Zeb2*, *Id2*, and *Tbx21* transcripts, which encode key regulators driving the terminal differentiation. The positive effect of CD160 on Tcf1 expression was further corroborated by the fact that the CD160+ cell displayed higher levels of SLAMF6 and downregulation of CD39 relative to CD160− cells, in line with a recent report that SLAMF6+ and CD39− are combined surrogate markers of Tcf1 expression during chronic viral infection (30). Given the prominent role of TCF1 in governing proliferative and survival CD8+ T cell pool (31, 32), we analyzed its expression in the adoptively transferred P14 CD8+ T cells after Clone 13 infection. The frequency of TCF1-expressing cells was notably higher in CD160+ subset than CD160− subset at both 11 and 27 dpi (Figure 3E). Thus, the gene expression signature of CD160+ CD8+ T cells is consistent with their proliferative and survival capability, indicating their potential as a long-persisting source for the functional pool of CD8+ T cells during chronic virus infection.

### Genetic Ablation of CD160 Resulted in Intrinsic Impairment of CD8+ T Cell Function During Chronic LCMV Infection

**FIGURE 4:**
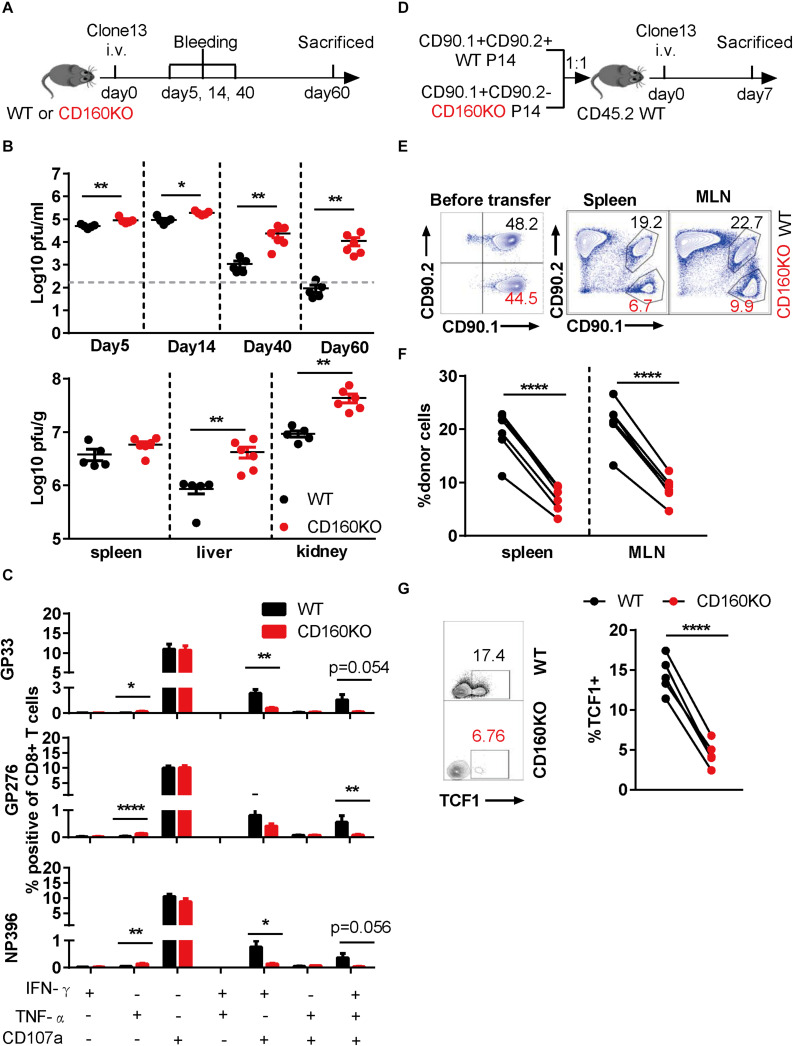
Genetic ablation of CD160 resulted in the intrinsic impairment of CD8+ T cell function during chronic lymphocytic choriomeningitis virus (LCMV) infection. **(A–C)** WT and CD160KO mice were infected with LCMV Clone 13 as scheduled in **(A)**; plasma viral loads were quantified on days 5, 14, 40, and 60 post-infection **(B)**, upper panel), and on 60 dpi the viral loads in spleen, liver, and kidney were also measured **(B)**, lower panel). **(C)** Summarized data showing the comparison between WT and CD160KO CD8+ T cells in the yield of IFN-γ/TNF-α/CD107a single-, dual-, and triple-positive cells after LCMV-specific peptide stimulation. Splenocytes were harvested and stimulated with LCMV glycoprotein peptide (GP33 and GP276) or LCMV nucleoprotein peptide (NP396) on 35 dpi. **(D)** Schematic diagram of adoptive transfer experiment to determine the nature of CD8+ T cell defect in chronic LCMV infection caused by CD160 deficiency. WT (CD90.1+ CD90.2+) and CD160KO (CD90.1+ CD90.2−) P14 CD8+ T cells were mixed at a 1:1 ratio (5,000 cells each), adoptively transferred into congenic (CD90.1–CD90.2+) naive recipient mice, and then infected with LCMV Clone 13. **(E)** Representative flow cytometric plots enumerating the abundance of WT and CD160KO donor cells before transfer (left) and in the spleen and mesenteric lymph node (MLN) of the recipient mouse on 7 dpi (right). **(F)** Pooled data of abundance assessment of P14 donor cells in the spleen (left) and MLN (right) of the recipient mice on 7 dpi. **(G)** Comparison of TCF1 expression by flow cytometry on adoptively transferred WT and CD160KO P14 cells isolated from recipient mice on 11 dpi. The data in **(B,C)** are analyzed by Mann–Whitney *U* test, and paired *t* test is used to analyze data in **(F,G)**. The error bars in **(B,C)** denote SEM. *P < 0.05; **P < 0.01; ****P < 0.0001.

To determine the functional role of CD160-expressing CD8+ T cell during chronic viral infection, we employed CD160 knockout (CD160KO) mice (19), which were previously used as *in vivo* model to study CD160 functioning in NK (19) and NKT cells (20). CD160KO mice along with WT mice were infected with LCMV Clone 13 for a period up to 60 days and subjected to analyses of viral loads and CD8+ T cells at various time points (Figure 4A). Compared to the WT group, the CD160KO group showed a significantly higher virus burden in plasma as early as on 5 dpi, and the gap was further expanded over time, reaching a 2-log difference by 60 dpi (Figure 4B, up). Similar trends were also observed for viral loads in the spleen, liver, and kidney measured on 60 dpi (Figure 4B, down). Thus, CD160 was essential for the effective control of chronic LCMV infection in mice. The virus-specific CD8+ T cell responses were evaluated on 35 dpi upon stimulation with either immune-dominant (GP33 and NP396) or sub-dominant (GP276) LCMV-specific synthetic peptide. For all the three peptides tested, reduced frequencies of elicited IFN-γ/CD107a dual-functional cells and IFN-γ/TNF-α/CD107a triple-functional cells were observed in CD160KO CD8+ T cells when compared to their WT counterparts, whereas no difference in IFN-γ or CD107a single-functional subpopulation was observed. Interestingly, an increase in TNF-α-positive population was also observed in CD160KO CD8+ T cells, though this represented a minor subset of functional CD8+ T cells (Figure 4C). Collectively, these results confirmed the requirement of CD160 for an optimal protective CD8+ T cell response during chronic LCMV infection.

To exclude the possibility that the CD8+ T cell impairment in CD160KO mice arises from a cell-extrinsic mechanism, we crossed P14 mice to CD160KO mice, which allowed us to obtain CD160KO P14 CD8+ T cells. Subsequently, we adoptively transferred equal numbers of WT P14 CD8+ T cells and CD160KO P14 CD8+ T cells into congenic mice, followed by infection with LCMV Clone 13 (Figure 4D). Notably, CD160KO P14 CD8+ T cells were significantly less than WT P14 CD8+ T cells at 7 dpi (Figures 4E,F). Furthermore, CD160KO P14 CD8+ T cells showed reduced TCF1 expression in comparison to their WT counterparts (Figure 4G), consistent with the previous observation made on CD160+ and CD160− wild-type cells (Figure 3E). Altogether these data added further evidences supporting that CD160 acts as a general promoter of proliferation/survival and functionality of CD8+ T cells during chronic infection in both human and mice.

## Discussion

Although the up-regulation of CD160 in chronic viral infection has been documented in many studies, its precise role remains controversial. An earlier report observed that a high baseline level of CD160 expression correlated with better response to ART (30), and Carolina Pombo’s study (25) found that the co-expression of CD160 and 2B4 defined the cytolytic CD8+ T cells in HIV-1+ elite controllers, suggesting that CD160 might play a protective role. In contrast, another study proposed that the co-expression of CD160 and PD-1 defined an advanced exhausted T cell population during chronic HIV-1 infection (24). The major caveat of this research is neglecting PD-1-CD160+ CD8+ T cells, which both previous studies and our study found to be the CD8+ T cell subpopulation distinguishing slow progressors from typical progressors. Here, using both chronic HIV and LCMV infection model, we sought to determine the actual linkage between CD160 and chronic viral infection. Our results proved that CD160 signaling positively regulates CD8+ T cells in a cell-intrinsic manner during chronic viral infection by demonstrating that (1) CD160 was positively correlated with CD4 counts and that CD160/PD-1 ratio could predict HIV slow progression status better than CD160 or PD-1 alone, (2) triggering CD160 on CD8+ T cells could enhance the degranulation of epitope-specific CD8+ T cells, (3) CD160+ CD8+ T cells were functionally superior to their CD160− counterparts in mice chronically infected with LCMV virus and exhibited a gene signature favoring proliferation/survival over terminal differentiation, and (4) CD160 knockout mice failed to effectively contain the LCMV virus. Our results highlight a critical and active role of CD160 signaling in host immunity against chronic viral infection. Notably, in line with our studies, two recent studies reported that CD160+ CD8+ T cells play positive roles in immune responses against malaria (33) and listeria (34).

CD160 may exert its actions on CD8+ T cells in several ways. First, upon up-regulation on CD8+ T cells after activation, the presence of CD160 may promote proliferative capacity and thereby expand the population of pathogen-specific CD8+ T cells and generate more effective T cells (including both effector and effector memory CD8+ T cells). Second, CD160 may facilitate the escape of CD8+ T cells from terminal differentiation, maintaining a pool of durable, polyfunctional cells during chronic virus infection. The expression of TCF1 and other gene expression signatures of CD160+ CD8+ T cells from LCMV chronically infected mice supported this view. Third, CD160 signaling may synergize with TCR signaling pathway to further enhance the cytotoxicity of CD8+ T cells by activating the PI3K–AKT and MEK–ERK signaling pathways. In accordance with its role, CD160 is dominantly expressed on PD-1-CD8+ T cells in HIV-infected subjects, suggesting that the large proportion of CD160+ CD8+ T cells are unlikely to be either regulated by PD-1/PD-L1 pathway or represent an exhaustive phenotype with advanced dysfunction. Indeed the majority of CD160+ CD8+ T cells also express HLA-DR and CD95, indicating that these cells are fully activated and readily available to exert their activities.

An earlier study showed that CD160 could bind to classic and non-classic MHC-I molecules and co-stimulate with anti-CD3 antibodies to sponsor a vigorous proliferation for activated CD8+ T cells (17). HVEM is the other known CD160 ligand/receptor reported to have a higher binding affinity for CD160 than MHC-I. However, using HVEM knockout mice, our preliminary analysis suggested that HVEM unlikely mediates the CD8+ T cell regulatory activity of CD160 (unpublished data). It is conceivable that the interaction between CD160 on CD8+ T cells and MHC-I molecules on APCs activates CD160 and its downstream pathway. This mechanism may be critical to ensure the appropriate activation of CD8+ T cells, as the CD28 co-stimulatory signaling is generally inhibited in the exhausted T cells by CTLA-4 as well as PD-1 (35). It is tempting to speculate that CD160 might take the place of CD28 to deliver the second signal to CD8+ T cells for effective activation. Indeed the observation that the ligation of CD160 by its antibody strengthened peptide-stimulated CD8+ T cell degranulation indicated that MHC-epitope might trigger both TCR and CD160 signaling pathways and thereby maximize CD8+ T cells functionality.

In summary, we identified CD160 primarily functioning as a costimulatory receptor molecule responsible for the optimal activation and proliferation/survival of CD8+ T cells, thus essential for virus control, during chronic virus infection. Our data suggest that CD160 may be a rational target for immune intervention in chronic virus infection as triggering CD160 is likely to further enhance the therapeutic effect of PD-1 blockade.

## Data Availability Statement

All datasets generated for this study are included in the article/Supplementary Material.

## Ethics Statement

The studies involving human participants were reviewed and approved by Ethics Committee of Shanghai Public Health Clinical Center. The patients/participants provided their written informed consent to participate in this study. The animal study was reviewed and approved by animal experiments were approved by Animal Care and Use Committee of authors’ institute, Shanghai Public Health Clinical Center.

## Author Contributions

JiX conceived the study. LXZ, AZ, and JuX performed the experiments and wrote the manuscript. CLQ provided data on CD4+ T cell counts. LGZ assisted in the microarray data analysis. YW provided data on viral loads. LXZ, AZ, JuX, JiX, CZ, and CQ analyzed the data. JiX and CZ revised the manuscript. All authors contributed to the article and approved the submitted version.

## Conflict of Interest

The authors declare that the research was conducted in the absence of any commercial or financial relationships that could be construed as a potential conflict of interest.

## Footnotes

1 https://david.ncifcrf.gov/

## Supplementary Material

FIGURE S1 Correlation between CD160 expression and parameters of chronic HIV-1 infection. **(A)** Frequencies of CD160+ PD-1+ cells among total CD8+ T cells in slow progressors (SP), typical progressors (TP), and HIV-1 seronegative controls (HIV−). **(B)** Association between CD160+ PD-1+ *versus* CD160-PD-1− CD8+ T cell subpopulation and CD4 counts (upper panel) or plasma viral loads (lower panel) in HIV-1-infected subjects. SP, *n* = 17; TP, *n* = 41. **(C)** Association between %CD160+ PD-1− CD8+ T cells (left column), %CD160-PD-1+ CD8+ T cells (middle column), CD160+/PD-1+ ratio (right column), and CD4 counts (upper row) or plasma viral loads (lower row) in TP group. *n* = 41. The calculated *p* and *r* values are presented in the corresponding figures. Data in **(A–C)** are analyzed by Spearman correlation and Mann–Whitney *U* test, respectively. The error bars in **(C)** represent SEM.

FIGURE S2 Fluorescence-activated cell sorting analysis of mouse IgG isotype control for the experiments shown in Figure 2A.

FIGURE S3 CD160 was upregulated on CD8+ T cells in HIV-1+ patients and defined an HLA-DR + CD95 + CD8+ T cell subset. **(A)** Representative flow cytometric plots of CD160 expression on four CD8+ T cell subsets: TCM (CD45RA-CCR7 +), T-naive (CD45RA + CCR7 +), TEM (CD45RA-CCR7−), and TEMRA (CD45RA + CCR7−). **(B)** Pooled data of flow cytometry-detected frequencies of CD160+ CD8+ T cells in the four CD8+ T subsets among three human groups: SP, slow progressor; TP, typical progressor; HIV-, HIV negative. **(C)** Representative flow cytometric plots assessing the co-expression of CD160 with CD38, HLA-DR, CD95, and CD127 on CD8+ T cells. **(D)** Summarized data showing a comparison of expression of CD38, HLA-DR, CD95, and CD127 between CD160+ and CD160-CD8+ T cells. Data in **(B,D)** are analyzed by Mann–Whitney *U* test and paired *t* test, respectively. The error bars in **(B,D)** denote SEM. ^∗^*P* < 0.05; ^∗∗^*P* < 0.01; ^∗∗∗^*P* < 0.001; ^****^*P* < 0.0001.

TABLE S1 Demographic and clinical characteristics of the human subjects in this study.
