# Supplementary figures and images for: CD160 Plays a Protective Role During Chronic Infection by Enhancing Both Functionalities and Proliferative Capacity of CD8+ T Cells
Source: Front Immunol. 2020 Sep 11;11:2188. doi: 10.3389/fimmu.2020.02188 (PMC7533580; doi:10.3389/fimmu.2020.02188)

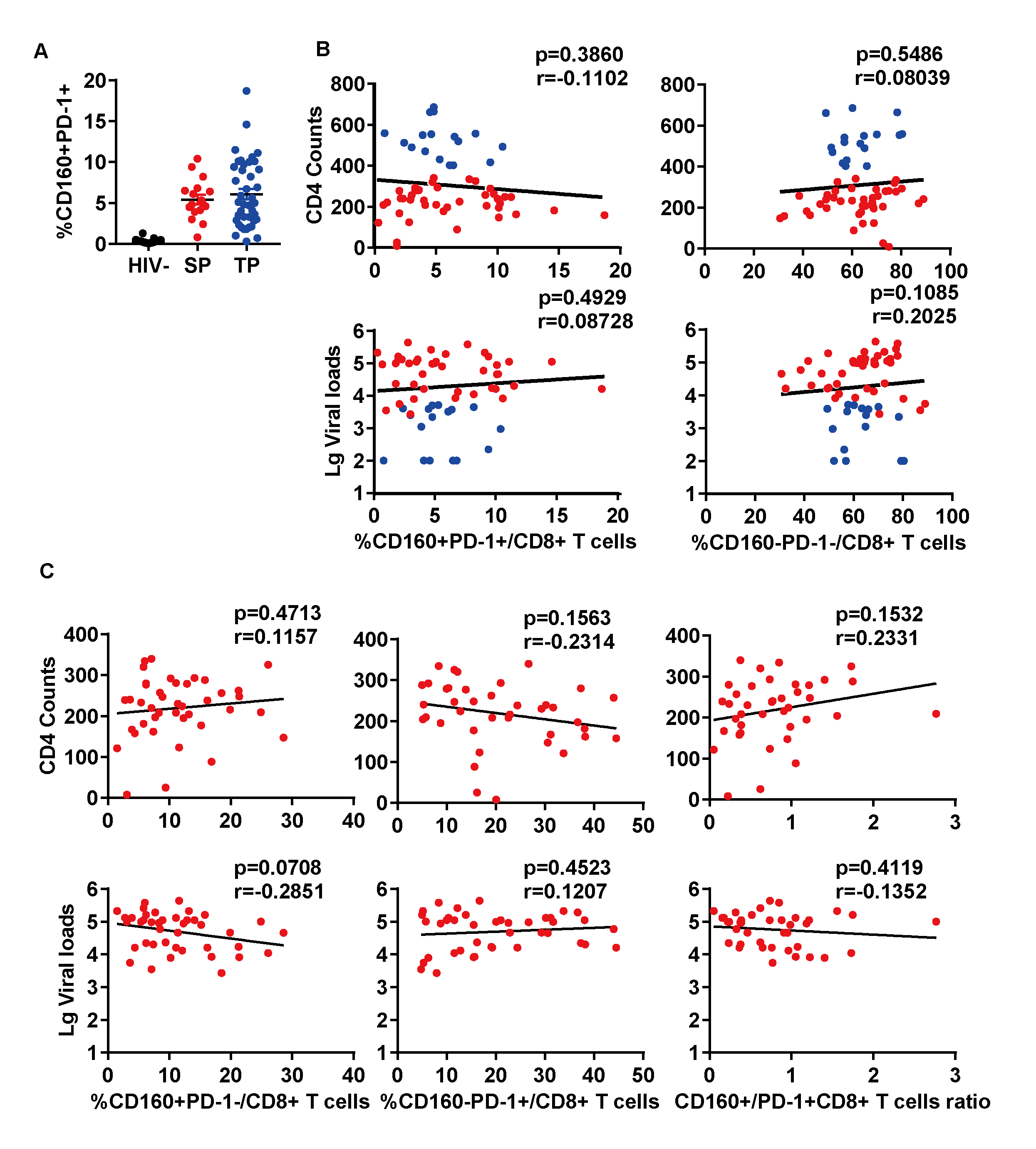

Supplement: FIGURE S1 — Correlation between CD160 expression and parameters of chronic HIV-1 infection. (A) Frequencies of CD160+ PD-1+ cells among total CD8+ T cells in slow progressors (SP), typical progressors (TP), and HIV-1 seronegative controls (HIV−). (B) Association between CD160+ PD-1+ versus CD160-PD-1− CD8+ T cell subpopulation and CD4 counts (upper panel) or plasma viral loads (lower panel) in HIV-1-infected subjects. SP, n = 17; TP, n = 41. (C) Association between %CD160+ PD-1− CD8+ T cells (left column), %CD160-PD-1+ CD8+ T cells (middle column), CD160+/PD-1+ ratio (right column), and CD4 counts (upper row) or plasma viral loads (lower row) in TP group. n = 41. The calculated p and r values are presented in the corresponding figures. Data in (A–C) are analyzed by Spearman correlation and Mann–Whitney U test, respectively. The error bars in (C) represent SEM. [file Image_1.tif]

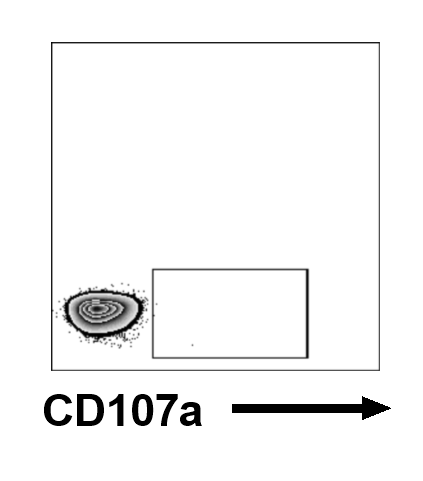

Supplement: FIGURE S2 — Fluorescence-activated cell sorting analysis of mouse IgG isotype control for the experiments shown in Figure 2A. [file Image_2.tif]

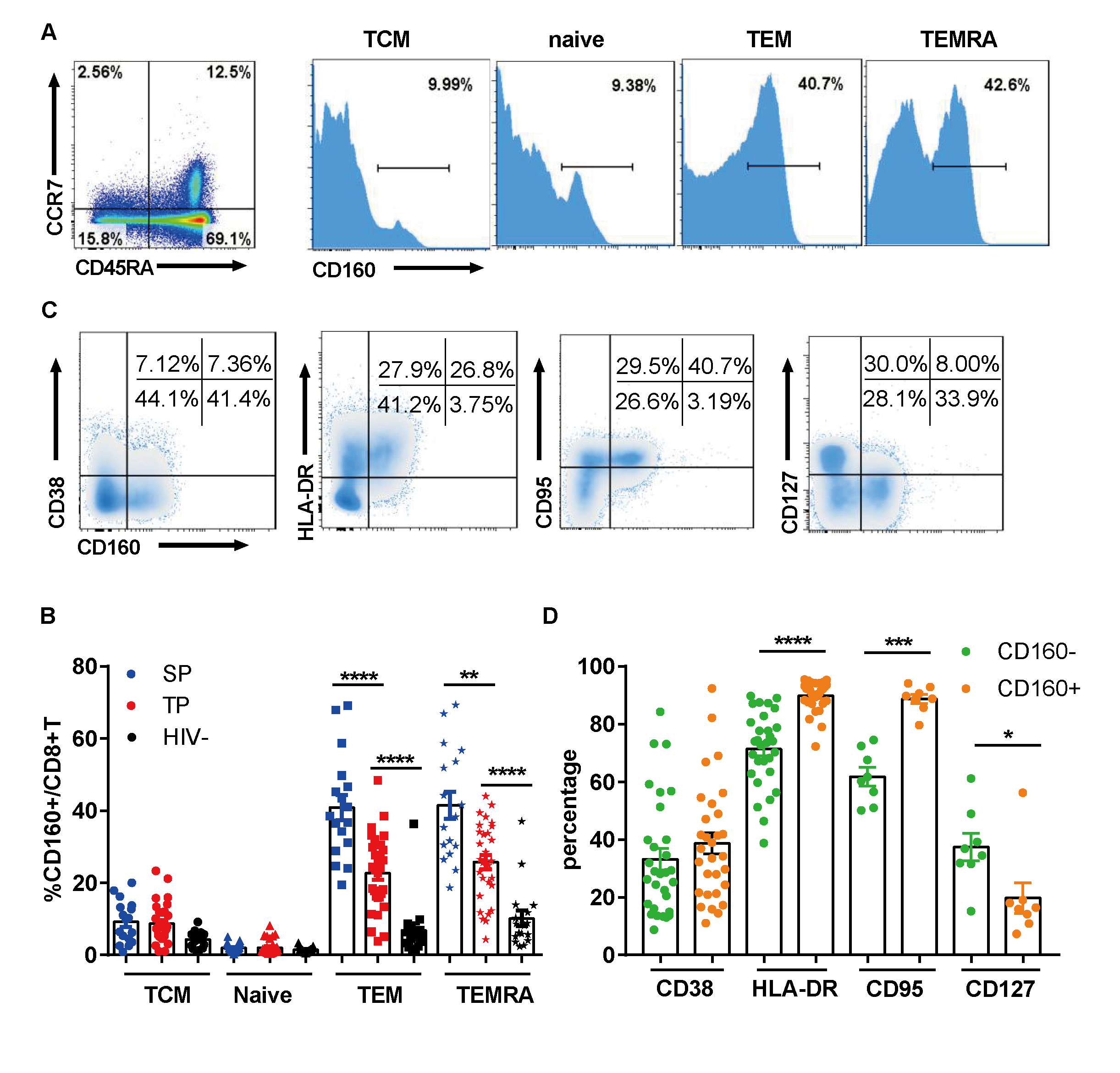

Supplement: FIGURE S3 — CD160 was upregulated on CD8+ T cells in HIV-1+ patients and defined an HLA-DR + CD95 + CD8+ T cell subset. (A) Representative flow cytometric plots of CD160 expression on four CD8+ T cell subsets: TCM (CD45RA-CCR7 +), T-naive (CD45RA + CCR7 +), TEM (CD45RA-CCR7−), and TEMRA (CD45RA + CCR7−). (B) Pooled data of flow cytometry-detected frequencies of CD160+ CD8+ T cells in the four CD8+ T subsets among three human groups: SP, slow progressor; TP, typical progressor; HIV-, HIV negative. (C) Representative flow cytometric plots assessing the co-expression of CD160 with CD38, HLA-DR, CD95, and CD127 on CD8+ T cells. (D) Summarized data showing a comparison of expression of CD38, HLA-DR, CD95, and CD127 between CD160+ and CD160-CD8+ T cells. Data in (B,D) are analyzed by Mann–Whitney U test and paired t test, respectively. The error bars in (B,D) denote SEM. ∗P < 0.05; ∗∗P < 0.01; ∗∗∗P < 0.001; ****P < 0.0001. [file Image_3.tif]
