# Supplementary material for: CD160 Plays a Protective Role During Chronic Infection by Enhancing Both Functionalities and Proliferative Capacity of CD8+ T Cells
Source: Front Immunol. 2020 Sep 11;11:2188. doi: 10.3389/fimmu.2020.02188 (PMC7533580; doi:10.3389/fimmu.2020.02188)
Supplement: TABLE S1 — Demographic and clinical characteristics of the human subjects in this study. [file Table_1.docx]

**Supplementary table 1. Demographic and clinical characteristics of human subjects in this study**

|  | **HIV-1 seronegative subjects (HIV-)** | **Slow Progressors**  **(SP)** | **Typical Progressors (TP)** |
| --- | --- | --- | --- |
| **Subject No.** | **33** | **21** | **46** |
| **Age , Mean (range)** | **42 (26-59)** | **44 (28-65)** | **44 (25-68)** |
| **Male** | **18** | **9** | **32** |
| **Female** | **15** | **12** | **14** |
| **CD4 counts(cells/ ul), Median (range)** | **NA** | **530 (402-818)** | **222 (15-340)** |
| **HIV-1 viral loads (copies/ml), Median (range)** | **NA** | **1887 (100-5000)**  **100=Undetectable** | **86080 (5500-430000)** |
| **HIV-1 infected time (year) (range)** |  | **9.6 (8-16)** | **5.3 (2-10)** |
