# Supplementary material for: CD160 Plays a Protective Role During Chronic Infection by Enhancing Both Functionalities and Proliferative Capacity of CD8+ T Cells
Source: Front Immunol. 2020 Sep 11;11:2188. doi: 10.3389/fimmu.2020.02188 (PMC7533580; doi:10.3389/fimmu.2020.02188)
Supplement: Supplementary file 5 [file Data_Sheet_1.docx]

**Supplementary Information**

**Antibodies and reagents used in flow cytometry**

Antibodies used for analyzing human CD8+ T cells included: anti-CD160-Alexa647 (clone: BY55), anti-PD-1-APC (clone: EH12.2H7), anti-CCR7-PerCP-Cy5.5 (clone: G043H7), anti-CD95-PB (clone: DX2), anti-CD107a-APC (clone: H4A3) were purchased from Biolegend; anti-CD38-APC (clone: HIT2) was from eBioscience; anti-CD3-PB (clone: UCHT1), anti-CD4-Amcyan (clone:SK3), anti-CD8-APC-H7 (clone: SK1), anti-CD127-PerCP-Cy5.5 (clone: HIL-7R-M21) were from BD Biosciences; anti-CD160-PE (clone: FAB6700P), anti-HLA-DR-ECD (clone: Immu-357) were from Beckman Coulter. Antibodies used for staining mouse cells included: anti-CD3-Percp-Cy5.5 (clone: 17A2), anti-CD8-PB (clone: 53-6.7), anti-CD8-Alexa488 (clone: 53-6.7), anti-CD90.1-APC (clone: OX-7), anti-CD90.1-BV711 (clone: OX-7), anti-CD90.2-PE (clone: 30H12), anti-PD-1-BV421 (clone: 29F.1A12), anti-CD160-PE (clone: 7H1), and anti-IFN-γ-PE (clone: XMG1.2) were from Biolegend; anti-CD90.2-PE-Cy7 (clone: 53-2.1) and anti-CD107a-Percp-eﬂuor710 (clone: 1D4B) were from eBioscience; anti-CD3-APC (clone: 17A2), and anti-TNF-PE-Cy7 (clone: MP6-XT22) were from BD Biosciences. The other used reagents included Live/Dead Near-IR Dead Cell Stain Kit (Invitrogen), EasySep Magnetic Human and mouse CD8+T enrichment kit (STEMCELL Technologies), and HIV-1 B clade Gag peptides pools, which were provided by NIH AIDS Program.
